# Working as frontline health facilitators, service providers, program supporters, and social health activists in Indian hilly terrain areas: A qualitative study of accredited social health activists’ experiences before and during the COVID-19 pandemic

**DOI:** 10.7189/jogh.12.05052

**Published:** 2022-11-29

**Authors:** Manisha Gore, Anand Kawade, Pam Smith, Hilary Pinnock, Sanjay Juvekar, RESPIRE Collaboration

**Affiliations:** 1 Symbiosis Community Outreach Programme and Extension, Faculty of Health Sciences, Symbiosis International (Deemed) University, Lavale, Pune, India; 2 KEM Hospital Research Centre, Vadu Rural Health Program, Rasta Peth, Pune, India; 3 Nursing Studies, School of Health in Social Science, NIHR Global Health Research Unit on Respiratory Health, University of Edinburgh, Edinburgh, United Kingdom; 4 NIHR Global Health Research Unit on Respiratory Health, Usher Institute, University of Edinburgh, Doorway 3, Medical School, Edinburgh, United Kingdom

## Abstract

**Background:**

Community health workers (CHW) contribute to achieving health targets of the Sustainable Development Goals (SDG) and Universal Health Care (UHC) in low- and middle-income countries (LMICs). In India, accredited social health activists (ASHAs) function as health facilitators, service providers, and programme supporters for rural and tribal communities and are at the frontline during the COVID-19 pandemic. We aimed to describe the ASHAs’ work roles both before and during the COVID-19 pandemic, explore the tasks ASHAs performed throughout the pandemic, and understand its effects on the evolving role of ASHAs.

**Methods:**

We used qualitative data from a pre-COVID-19 study conducted in 2018-2019 including face-to-face interviews with purposively sampled ASHAs and their health care supervisors (n = 18) from rural Maharashtra state (India), and a follow-up study during the COVID-19 pandemic using telephonic interviews with a subset of participants from the pre-COVID-study (n = 8). Data were analysed thematically using MAXQDA v11.00.

**Results:**

The primary theme in the pre-COVID-19 study was ASHAs’ role as described above, except as social health activists, linking beneficiaries to the local maternal and child health care services, distributing medicines for common illnesses, access to government schemes, and engaging in multiple health surveys. During the pandemic, raising awareness, screening of at-risk populations, arranging referrals, providing treatment and follow-up to COVID-19 patients, and supporting their family members. These activities increased the workload and health risks to ASHAs and their family, causing stress and tension among them. However, they had effectively carried out the new duties. ASHAs have improved their status, earning praise from families, society, and the government. They were honoured with the Global Health Leaders Award at the 75th World Health Assembly.

**Conclusion:**

ASHAs’ contribution to the health system improved the indicators related to maternal and child health during the pre-COVID-19 pandemic. Additionally, they maintained frontline health care during the COVID-19 pandemic, demonstrating resilience despite the challenges of increased workload and stress. However, the COVID-19 pandemic highlights the need to respond to and understand the implications of ASHAs’ evolving roles.

Although the community health workers (CHWs) programme started in China in 1930, the concept has been expanded to help achieve health targets of Sustainable Development Goals and Universal Health Care in low- and middle-income countries (LMICs) [1-4]. While Bangladesh, Brazil, and Nepal succeeded in reducing under-five mortality through robust CHW programmes [5], those in Pakistan, Ethiopia, and Malawi were focused on family planning and maternal and child health [1]. Additionally, in India, they contribute to the prevention, identification, and treatment of non-communicable diseases (NCDs) [6,7], including chronic obstructive pulmonary disorders (COPD) and asthma, like initiatives in Uganda, Kyrgyzstan, and Malaysia [8]. India introduced CHWs in the early 1970s through small projects in specific areas of the country [9]. Such projects showed that CHWs could be an asset for India’s public health system through the Mandwa Project in Maharashtra [10], the over-five-decade-old Vadu Rural Health Program (VRHP) [11], the Society for Education, Welfare and Action (SEWA) Rural project in Gujrat [12], and the Mitanin CHW Program of Orissa [1]. Nationwide CHWs were introduced after the declaration of Alma Ata as community health volunteers (CHVs) and (more recently) as accredited social health activists (ASHAs) (ie, village women selected and trained to work as ASHAs); providing health care services to underprivileged, poorly literate, culturally diverse, and hard-to-reach rural/tribal Indian communities [1,8,9]. Their roles and responsibilities, mandated by operational guidelines [8], have evolved over time. ASHAs work as health educators and promoters, linking workers (between population and government health care services) and service providers [8], raising health awareness (nutrition, sanitation) to address barriers to health care, and participating in surveillance [13].

The COVID-19 pandemic affected CHWs’ roles in many LMICs [14-17]. In Kenya, Mali, Malawi, and Uganda, CHWs were supported to maintain the pace and scope of community-delivered care during the pandemic [15]. CHWs in Nigeria (and even in high-income countries like Texas) reported exhaustion due to increased workload [14-16].

We previously explored the ASHA role in a rural area of Maharashtra state, India [18], concluding that ASHAs perceived themselves as voluntary community health workers rather than health activists. During the COVID-19 pandemic, they were given multiple additional responsibilities of screening, identifying high-risk populations, referring patients to treatment centres, monitoring isolated patients, and supporting families of people with COVID-19 [13]. There was limited training before taking on these new roles, and despite the increased workload, there were no systems for timely remuneration. [13]. The lack of personal protective equipment meant that ASHA and their families were contracting COVID-19. We thus investigated ASHAs’ activities as front-line health workers during the pandemic and returned to our earlier work to better understand the impact of the pandemic on evolving roles of ASHA compared to their pre-COVID roles as health facilitators, service providers, programme supporters, and social health activists.

## METHODS

We synthesized data from two sources. The first source was a mixed-methods pre-COVID-19 study, conducted between September 2018 and March 2019 [18]. Following the start of the COVID-19 pandemic (July to September 2021), we contacted all 18 participants from the qualitative pre-COVID-19 study and requested a follow-up qualitative interview exploring the pandemic’s impact on ASHA roles. The most common reason for non-participation was being too busy with pandemic duties (eg, conducting immunization sessions).

### Data collection

Two researchers (a qualitative researcher and an epidemiologist/health scientist) conducted in-depth face-to-face interviews in the pre-COVID-19 study. The interviews undertaken during the COVID-19 pandemic were conducted and audio-recorded over the telephone (to comply with social distancing regulations) [19] by MG, a female anthropologist who analysed the data for both studies. The interviews took place in the evenings, when the participants could dedicate sufficient time and were not constrained by work obligations, and lasted from 60 to 90 minutes. No ethical issues arose. Interview guides had questions on roles and responsibilities during the pandemic, risk perception, the experience of tension and stress, coping mechanisms, and the pandemic’s effect on their roles and responsibilities. Interviews were conducted in Marathi, transcribed verbatim manually, and translated into English by MG.

### Analysis

MG and SJ (using MAXQDA v11.00) (re)analysed the pre-COVID-19 study and the study conducted during the pandemic to determine the specifics of the roles and the challenges experienced in fulfilling them. They repeatedly examined the transcripts using an inductive approach to uncover frequently reported patterns connected to objectives with similarities and differences. We coded the data from both studies, formed categories, and identified themes. We then compared the data related to the four defined ASHA roles (health care facilitator, service provider, health activist, and programme supporter) between the two studies to identify changes in the perceived role influenced by the pandemic.

## RESULTS

### Interviewee characteristics

**Figure 1:**
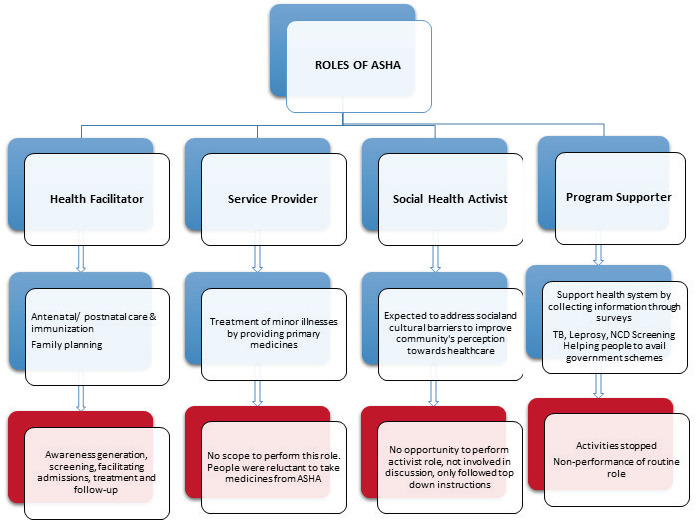
Roles of ASHA.

**Figure 2:**
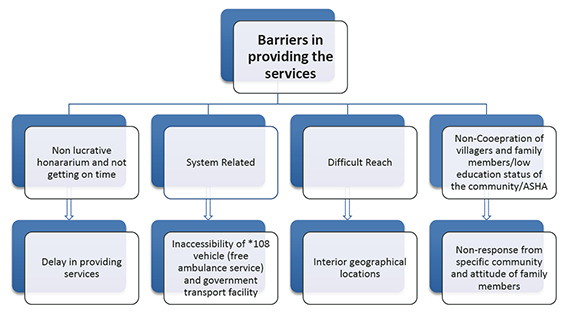
Barriers to providing the services. *108, Dial-108, or one-zero-eight is a toll-free telephone number for emergency services in India. It is currently operational in 18 states. The 108 Emergency Response Service is a free emergency service providing integrated medical services.

**Figure 3:**
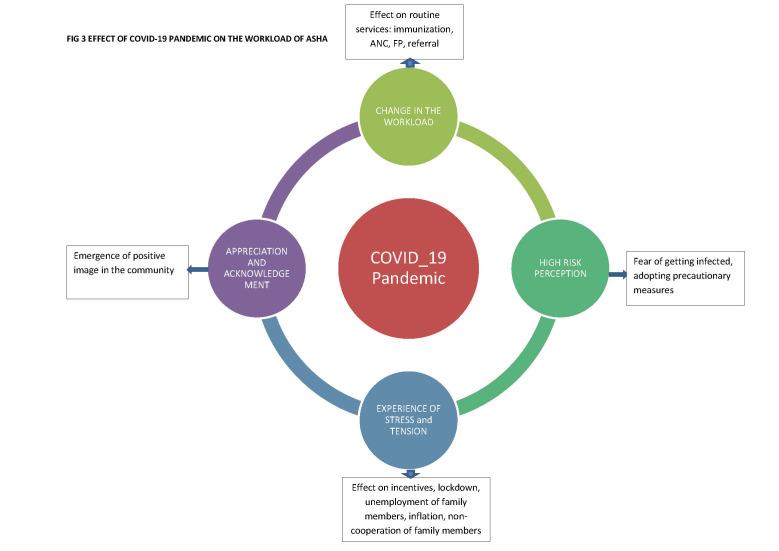
Effect of COVID-19 pandemic on the workload of ASHAs.

The eight ASHAs (all female) interviewed in the pre-COVID-19 study had eight to 10 years of work experience and two had >10 years of experience. Six of the ASHA (three each from rural and tribal areas) were recruited to the study conducted during the pandemic. Of the four block facilitators (BFF), two (one each from rural and tribal areas; all female), were interviewed in the pandemic-period study. The auxiliary nurse midwives (ANMs) were more senior with one having over 25 years of experience (**Table 1**). We present our findings in thematic maps (**Figure 1** and **Figure 2**) and a framework (**Figure 3**) to answer the three study objectives. **Table 2** explains the local terms used by the participants and **Table 3** shows the examples of verbatim to showcase accredited social health activists’ experiences during the pre-COVID-19 and COVID-19 phases.

**Table 1:** Demographic information of study participants

IDI	Age (years)	School (standard)	Experience (years)	COVID-19 time study
A01	30-39	8-10	8-10	AFU05
A02	40-49	11-12	8-10	None
A03	30-39	8-10	8-10	AFU06
A04	30-39	8-10	8-10	AFU01
A05	30-39	8-10	8-10	AFU02
A06	30-39	8-10	8-10	None
A07	40-49	8-10	>10	AFU03
A08	30-39	8-10	>10	AFU04
**Key informants**				
BFF01	30-39	>12	<8	BFFFU01
BFF02	30-39	11-12	8-10	None
BFF03	30-39	>12	<8	None
BFF04	30-39	11-12	8-10	BFFFU02
ANM01	50-55	8-10	>25	None
ANM02	30-39	11-12	<11	None
ANM03	40-49	>12	<11	None
ANM04	50-59	8-10	1-5	None
MO01	30-39	>15	1-5	None
MO02	40-49	>15	<10	None

ASHA – accredited social health activist, BFF – block facilitator, ANM – auxiliary nurse midwife, MO – medical officer

**Table 2:** Explanation of local terms in Indian context

1.	GP is village self-government. It is a democratic framework at the grass-root level, a political organization that serves as village cabinet.	www.nrega.nic.in
2.	Sarpanch, also known as “Gram Pradhan” or “Mukhiya”, is a decision-maker, elected by the Gram Sabha (village-level constitutional body) of GP. Gramsevak is GP's secretary, responsible for GP's functioning, accounts, and records.	www.pria.org
3.	Two-wheeler riders: Villagers use two-wheeler vehicles to facilitate routine commuting. ASHA frequently accompanies such persons for household visits in hilly locations to make travel easier.	Not applicable
4.	Aadhaar card with a 12-digit random number, issued by Unique Identification Authority of India UIDAI (Authority) to Indian residents after satisfying verification process laid down by Authority. Any individual, irrespective of age and gender, who is a resident of India, may voluntarily enrol to obtain Aadhaar card. Pan Card – Permanent Account Number is a ten-character alphanumeric identifier, issued in the form of a laminated “PAN card”, by Indian Income Tax Department, to a “person” who applies for it or to whom the department allots the number without an application. It can also be obtained as PDF file.	www.uidai.gov.in, www.incometaxindia.gov.in
5.	Pradhan Mantri Matru Vandana Yojana (PMMVY) is a government-sponsored direct benefit transfer scheme with a cash incentive of ₹5000provided directly in the bank/post office account of Pregnant Women and Lactating Mothers.	www.wcd.nic.in
6.	Janani Suraksha Yojana is a government-sponsored scheme which integrates cash assistance for mothers with delivery and post-delivery care.	www.nhm.gov.in
7.	Shardagram Arogya Sanjeevani Program- medicines and nutrition supplements are provided to ANC.	www.nhm.gov.in

GP – Gram Panchayat, ASHA – accredited social health activist, ANC – antenatal care

**Table 3:** Examples of verbatim to showcase accredited social health activists’ experiences during the pre-COVID-19 and COVID-19 phases

No.	Theme	Verbatim
1.	Role of ASHA as Healthcare facilitator	“One night, a woman from a remote hamlet went into labor. She experienced pains all night, and the next morning, I received a message from her family stating that she would deliver at any time. I rushed to her house and delivered her baby. I was new at that time and had little experience, yet I had the courage to perform” (ASHA-X; tribal).
		“I took a woman requiring antenatal care to hospital and returned at midnight as I had to send my children (to school) early next morning” (ASHA-X; rural).
2.	Barriers to performing the role of a service provider	“I have spent out-of-pocket to take the patient to PHC for a surgery because he was unable to afford the transportation though managed to get the PHC vehicle while returning” (ASHA-X; rural).
3.	Villagers’ non-cooperation	“Even if I visit 1-2 times, I develop rapport with the community, ASHA lacks these skills and that is one reason why ASHA fails to fulfil duties”. “It’s not just the villagers but people living on farms that need to be visited and who often are missed by ASHA” “Such instances increase non-cooperation from the villagers” (ASHA-X; rural).
4.	Experiences during generating awareness in the community during COVID-19	“At first, people did not open their doors for us but as they became aware of the situation through WhatsApp etc., they cooperated” (ASHA-X; rural).
		“I was scared very much to do the household visits, but soon after ANM talked to me, I realized that this is the best opportunity to prove ourselves” (ASHA-X; tribal).

ASHA – accredited social health activist, ANM – auxiliary nurse midwife

### Accredited social health activists’ role during the pre-COVID-19 study

#### Healthcare facilitator

ASHAs serve as link workers, ensuring that women receive antenatal (ANC) and intrapartum care. This entails making regular home visits to young couples, registering newlyweds, and identifying and registering pregnant women for ANC. ASHAs provide health education, dispense iron and folic acid tablets, and escort pregnant women to the primary health centre (PHC) for antenatal checks. They usually accompany women in labour to a government health facility for a safe birth and stay with her when necessary. The participants perceived this as fulfilling work; however, it requires great mental focus. ASHAs visit new mothers on alternate days to support new-born care, disseminate family planning information, and assist auxiliary nurse midwives (ANMs) with post-natal care (recording blood pressure and arranging investigations). They perform the dual responsibilities of educators and link workers in identifying and bringing eligible children to immunization camps, while also working on mobilizing the villagers and escorting them to the PHCs for health-related issues.

#### Service provider: First point of health care

As the first contact for health care in the villages, ASHAs are trained to treat minor illnesses and perform other tasks (for example, monitor blood pressure). They provide directly observed therapy (DOTS) to tuberculosis (TB) patients and hold stocks of medicines (for example, oral rehydration solutions, iron and folic acid, chloroquine) which they dispense as required. Being residents of the same village, they can deliver accessible, timely, and cost-effective health services in remote places with limited communication/transport facilities and to a population with high illiteracy rates. The MO and BFF considered that ASHAs considerably reduced the health system’s workload, as they manage minor ailments and bring patients to the local health facility. A BFF corroborated this opinion, stating that ASHAs in tribal regions “do a great job”, serving fewer beneficiaries than their rural colleagues.

#### Barriers to performing the service provider role

Barriers (**Figure 2**) included problematic systems, difficult geographical reach, and delayed and non-lucrative honorariums.

#### Problematic systems

There were serious problems with public transport. Delay in attendance of the free health care emergency ambulance (“108”) was a major concern. ASHAs cited instances when delayed transport had resulted in home delivery. A vehicle is stationed at each PHC but is often not available during an emergency due to a driver not being available, a lack of fuel, or because the single vehicle was already attending to a patient in another village. A few ASHAs mentioned how they organized vehicles by paying “out-of-pocket”.

#### Difficult to reach

Limited transport for reaching distant villages/hamlets hampered the ASHAs’ work and could adversely affect services. Pregnant women living in hilly areas must trek down dangerously steep slopes for ANC. If ASHAs could not be accompanied by two-wheeler riders to remote locations, they restricted visits to houses within walking distance. ANM understood the challenges if pregnant women in remote villages were not visited. An ASHA’s region constitutes a wadi (hamlet) consisting of 15-30 houses (preferably the village they live in). Therefore, they have demanded that bicycles should be provided to ASHAs travelling more than three kilometres a day to help with transport.

#### Villagers’ non-cooperation in certain situations

Several examples were given of situations where villagers mistrusted or did not cooperate with ASHA services. People with TB often refused to attend follow-up visits. The Kathkari tribal community did not respect ASHAs, believing that (unlike doctors) they lacked knowledge. The villagers were suspicious when ASHAs asked for their bank details for the transfer of scheme funds. They did not disclose the information, and as a result, ASHAs were not eligible for the incentives. Additionally, language could be a barrier if ASHAs did not belong to the same community, especially in Thakar tribes, though some ASHAs had learned the dialect.

### Social health activist role

ASHAs did not describe themselves as “health activists” because they thought of themselves as “health workers”. They addressed health-related issues but had no voice against the socio-cultural barriers that prevent communities from accessing health care services. They were unhappy that they were ignored by MO and ANMs during discussions on strategies to improve the health status of the villagers.

### Program supporter

ASHAs supported health programmes by conducting multiple surveys, including family planning, welfare, leprosy, TB, cancer, oral health, dengue, malaria, domestic water storage practices, and identification of people with disabilities. They facilitate government schemes (such as Pradhan Mantri Matru Vandana Yojana benefits) available to the community. ASHAs reported how they opened bank accounts for villagers, which could be a long process if the necessary documentation was lacking.

### Accredited social health activists’ role during the COVID-19 pandemic-period study

ASHAs were trained online two to three times by the MOs during the first wave of the pandemic, followed by face-to-face training in small groups at the PHCs. However, some ASHAs initially felt “completely ignorant” about (for example) the use of a pulse-oximeter and thermometer, which caused some confusion until they received guidance from ANMs.

### Healthcare facilitator

#### Generating awareness

During the first wave of the COVID-19 pandemic, ASHAs played a crucial role in raising awareness among the villagers by undertaking surveys and facilitating contact with hard-to-reach and remote populations. Use of social media platforms such as WhatsApp groups helped disseminate information. Disease prevention communication materials were displayed in public places (bus stops, schools, temples, Gram Panchayat offices). For tribal communities, information pamphlets were read out. Rickshaws/other vehicles with speakers disseminated vital information. The Gram Panchayat raised funds to pay for these intensive awareness campaigns and preventative measures such as disinfecting villages twice a week and distributing hygiene kits containing soap, sanitizers, and masks. Initially, ASHAs were apprehensive about making the visits but agreed to complete duties after discussion with BFF and/or ANM. When ASHAs visited a residence to undertake the survey, a few reluctant villagers asked many questions, refused admittance out of fear of infection, insulted them, or behaved badly. A quote from an ASHA in a rural area speaks a thousand words (**Table 3 **).

#### Screening the population to identify COVID-19 patients

The primary task for ASHAs throughout the pandemic was screening villagers for COVID-19 symptoms. ASHAs visited every household and enquired about any family member suffering from symptoms of cold, cough, fever, headache, or body pain. People showing mild symptoms were checked for fever (using thermometers) and oxygen saturation (using pulse oximeters). Anyone suspected of having COVID-19 was referred for testing, and if positive, treated at COVID-19 care centres. ASHAs undertook stringent tracing of the positive person’s contacts in the last 10 days, checking for patterns of movement, travel history, and so on. Family members were tested, as well, with those testing positive joining the patient in COVID-19 care centres, and those who were negative being confined to home and instructed not to visit any public place for the next 14 days. ASHAs monitored family members for COVID-19 symptoms, checked temperature and oxygen saturations, counselled families about preventive measures, and provided basic medicines (paracetamol, multivitamins). They also supported the families by delivering essentials during quarantine, leaving bags of groceries and vegetables outside the houses for family members to pick up.

The ASHAs’ target was screening 100-150 people every day. Walking/traveling to distant houses took additional time, so the target could not always be achieved. Some ASHAs devoted four to six hours daily to this survey, visiting flexibly as per villagers’ availability. The workload increased for ASHAs who had higher numbers of COVID-19 patients in their respective villages. A few monitored more than 100 patients, along with patients’ family members. ASHAs with fewer COVID-19 patients and ANM/BFF were deputed to assist them. If people had to go outside the house or to any public place, people reported this to ASHAs. A team of ANM/MO/Sarpanch/Gram Sevak made surveillance visits to the homes of people under quarantine and sprayed disinfectants in the surrounding areas. ASHAs reported working eight to 12 hours a day during the first pandemic wave (February to November 2020) as they managed frequent enquiries, monitored people with mild symptoms, or quarantined and screened people in high-risk groups (such as people with hypertension, diabetes, and the elderly and small children). The workload was further increased by their routine tasks.

#### Continued work despite perceived high-risk of contracting COVID-19

Despite apprehension about engaging in the screening survey, after discussions with the BFF and ANM, ASHAs accepted that it was their role as a frontline health worker. Fear of infection, trepidation, and uncertainty were initial emotions, but they later gained confidence in making field visits. The families of a few ASHAs were hesitant about letting them undertake the screening, but the BFF intervened to explain the gravity of the situation and the importance of the work. Four ASHAs ignored their family opposition to continue work during the pandemic, but one left the role. ASHAs over 50 years were exempt from work, but all preferred working. The fear reduced gradually, especially after vaccination. ASHAs believed that they were serving people and would be protected by God. They followed precautionary measures, asking information from a safe distance and phoning (instead of visiting) houses with children. Preventive methods included wearing N95 masks, scarves, and gloves, using sanitizer after touching surfaces, and bathing with hot water after returning home. Some isolated themselves in different rooms before entering home after returning from work. ASHAs limited the time spent in homes, but when residents had questions or concerns, they were given appropriate attention. Despite taking preventive measures, one ASHA contracted COVID-19.

#### Impact of COVID-19 on accredited social health activists’ roles

The pandemic changed the health situation in rural and tribal areas and affected ASHAs’ roles and responsibilities (**Figure 1**). The routine work stopped, as they focused on pandemic-related activities. The health care facilitator role, involving extensive pandemic-related work, dominated their other three roles (see above). ASHAs, ANMs, and MOs unanimously reported that routine ASHA responsibilities, such as antenatal services, child immunization, and family planning work, were hampered. Pandemic restrictions meant that women could not travel to health care facilities to access pregnancy services. Family planning camps were cancelled and put on hold for a year. Child immunization was maintained, but poorly attended. Many women started bringing children when the restrictions eased which delayed doses but avoided omissions. At times, people hesitated to approach ASHAs, considering them to be at high risk of COVID-19 because of their work. Depending on their socioeconomic level, some people approached private physicians, while others approached government hospitals. During this time, the PHCs were open and provided treatment.

#### Stress and tension because of the pandemic situation and change in the roles

ASHAs were stressed during the pandemic for various reasons, including the substantial increase in work hours and shift in work patterns (as the pre-pandemic routine work was replaced with new, unpredictable COVID-19 related tasks) and the perceived high risk of contracting COVID-19 and transmitting it to family members (**Figure 1**). The cessation of routine work affected the ASHAs’ incentives and created financial tension, until the government authorized payments without monitoring routine work, which helped relieve the stress. Even then, some ASHAs complained they did not receive the honorarium for four to five months. Insufficient remuneration, delayed payment, unemployment of family members, lockdown, and inflation forced ASHAs to engage in other income-generating activities, including farming and daily waged work. Initially, Anganwadi sevika and schoolteachers assisted; later, ASHAs shouldered the tasks alone. BFFs helped ASHAs by providing precise instructions regarding their tasks, accompanying them on visits, and assisting in counselling resistant people, which helped relieve stress.

#### Recognition, acknowledgement, and appreciation

ASHAs’ image improved positively as people witnessed their exhaustive efforts and proactive approach to solving challenges and extending selfless support to COVID-19 patients. They received love and acknowledgement for their diligent follow-up when the movements were restricted. People expressed their concern about ASHAs’ health, advising them to “take good care”.

## DISCUSSION

### Summary of the key findings

This paper synthesizes the findings of two studies conducted before and during the COVID-19 pandemic. Before COVID-19, ASHA performed in three of their four defined roles: as “Health facilitators”, “Service providers”, and “Programme supporters”. They were task-driven and not enabled to take on the role of “Health activist”. The COVID-19 pandemic resulted in a dramatic shift in their responsibilities as the “Health care provider and facilitator” role took over all other tasks. There was an increase in workload, experiences of stress and anxiety, and a backlog of routine tasks, but they received widespread appreciation for their frontline activities.

### Strengths and limitations

In the pre-COVID-19 study, the multi-stakeholder perspective (ie, interviews with ASHAs, ANMs, BFFs, and MOs) enabled a comprehensive understanding of ASHA work and roles. Data collection was iterative, as participants’ views, opinions, and experiences informed subsequent interviews. Telephone interviews with ASHAs in the COVID-19 study were scheduled in the evenings when ASHAs were at home, leaving time to explore their experiences in detail. Researchers experienced connectivity issues with remotely located ASHAs, which disrupted and extended interviews. Interviews were conducted in Marathi, the language used by ASHAs in rural and tribal areas. Prolonged engagement (six of eight ASHA in the pre-COVID-19 study [17] participated in the COVID-19 study) and a feedback meeting after the pre-COVID-19 study improved trustworthiness,[20], though pandemic workload precluded MOs and ANMs from being involved in the COVID-19 study, which could have led to possible bias. Interpretation by a multi-disciplinary research team enhanced credibility. We maintained reflexivity throughout the study, as detailed notes and researchers’ biases were documented and discussed within the team [21].

### Discussion in relation to other literature

#### Pre-COVID-19 roles and barriers in performing the service provider duties

During the pre-COVID-19 study, our findings on the four defined ASHA roles echo those of other studies. The role of the “Health facilitator” is confirmed in studies in Karnataka and Manipur [22,23], and ASHAs in Udipi were highly aware of this role [24]. The tasks of frontline “Service providers” (treating minor illnesses or monitoring blood pressure) were well documented in studies from rural Manipur [23], though were less commonly provided in Karnataka [22]. The role of “Programme supporters” was also described in Karnataka, where the ASHA role was perceived as predominantly that of a link-worker/facilitator [23]. The lack of the “Health activist” role that we observed in our pre-COVID-19 study is reflected in other studies across India [18,23]. The reasons for this warrant further research, but may include a health system’s lack of awareness and (consequently) failure to prepare ASHAs for an “activist” role. Barriers influencing ASHAs’ performance included delayed and scanty honorariums, health care system flaws regarding the operation of the “108” emergency vehicle, ASHAs’ training for the “Activist” role, geographical remoteness, and time spent travelling. Several studies have reported that ASHAs are unhappy with the present incentive-based honorariums and are seeking permanent jobs with salaries [22,24]. Non-availability of transport has been described in other studies [19,25], for example, emergency “108” vehicles were often delayed in Orissa [25]).

#### Impact of the COVID-19 pandemic on ASHA roles and well-being

Our finding that the pandemic resulted in an unforeseen shift in roles is supported by studies from other LMICs, summarised in a review article [26]. In many countries, CHWs were engaged in COVID-19-related tasks, including raising awareness about the disease, implementing precautionary measures, contact tracing, coordinating patient care, providing support, and helping overcome stigma at the community level [26,27]. As in our study, this disrupted their routine work.

Stress and tension amongst CHWs due to COVID-19 restrictions and fear of contracting COVID-19 have been reported (for example, among CHWs engaged in door-to-door surveys in Texas, USA) [22,27], but our study extends these findings with an in-depth exploration of ASHAs’ experience of the changed responsibilities and increased workload [27]. There is evidence of stress in frontline health care professionals, including clinicians, nurses, and paramedical employees, but a few include CHWs [28].

#### Understanding the evolving roles through the lens of theories

Role identity theory has been used to examine CHWs' perceptions, experiences, and identities as they undertook new roles (eg, in rural South Africa [29]). This theory, [30] describes the process through which individuals assign meaning to themselves in the context of their professional roles. A complex mix of community “insider,” “outsider”, and “broker” identities were assumed by CHWs in response to the diversity of requirements and expectations from their role; each identity offering a distinctive way for the CHW to place themselves in the community. During the pandemic, ASHA perceived both “insider” and “outsider” role identities, as they provided services to their own community as an “insider”, but were considered “outsiders” when people were suspicious of them as government health care personnel. Similarly, ethnographic accounts of CHWs in 21st-century Africa, Latin America, and Asia reflect their involvement in significant social, political, and economic developments, as well as in pursuing improvements in their local community [31].

Current CHW roles are aimed at community management of disease/health, technical functions, and reporting to authorities, and typically underplay the activist role [18,31]. In a study from South Africa, CHWs performed diverse health and social care professional roles and felt as if they contributed to the health system [32], finding purpose in their jobs from the community’s positive response and associations with team leaders. Learning from this approach might support the Indian government to adapt and strengthen ASHAs’ roles and responsibilities. To understand the nuanced changes in the CHWs' roles both nationally and internationally, future research in this area is crucial.

## CONCLUSIONS

Health services globally were disrupted by the COVID-19 pandemic, including in India [18], as limited resources were diverted to the management of the pandemic [13] and the population avoided accessing routine health care because of fear of infection. During this time, India’s resilient ASHAs maintained frontline health care, despite increased workload and stress. Their award as Global Health Leaders [13] formally acknowledges their contribution at the highest levels. ASHAs’ long-term role in improving health indicators (especially related to maternity and child health [33]) is well recognized, but the pandemic has highlighted the need to understand evolving ASHA roles and address the persistent challenges. The foremost practical requirements are to ensure their financial security, a robust system for regular payment of honorariums, provision of adequate resources, continuous supervision with training and capacity building in areas of public health. The pandemic has highlighted the potential for strengthening the “Social activist” role. These strategies could secure the sustained and multidimensional role of ASHAs, enabling their ongoing impact on the health of vulnerable populations of India.
